# Estimation of aortic stiffness by finger photoplethysmography using enhanced pulse wave analysis and machine learning

**DOI:** 10.3389/fcvm.2024.1350726

**Published:** 2024-03-11

**Authors:** Henrik Hellqvist, Mikael Karlsson, Johan Hoffman, Thomas Kahan, Jonas Spaak

**Affiliations:** ^1^ Division of Cardiovascular Medicine, Department of Clinical Sciences, Danderyd Hospital, Karolinska Institutet, Stockholm, Sweden; ^2^ Marcus Wallenberg Laboratory for Sound and Vibration Research, Department of Engineering Mechanics, KTH Royal Institute of Technology, Stockholm, Sweden; ^3^ Division of Computational Science and Technology, KTH Royal Institute of Technology, Stockholm, Sweden

**Keywords:** photoplethysmography, arterial stiffness, vascular ageing, machine learning, prediction models, pulse wave analysis, pulse wave velocity wearables

## Abstract

**Introduction:**

Aortic stiffness plays a critical role in the evolution of cardiovascular diseases, but the assessment requires specialized equipment. Photoplethysmography (PPG) and single-lead electrocardiogram (ECG) are readily available in healthcare and wearable devices. We studied whether a brief PPG registration, alone or in combination with single-lead ECG, could be used to reliably estimate aortic stiffness.

**Methods:**

A proof-of-concept study with simultaneous high-resolution index finger recordings of infrared PPG, single-lead ECG, and finger blood pressure (Finapres) was performed in 33 participants [median age 44 (range 21–66) years, 19 men] and repeated within 2 weeks. Carotid–femoral pulse wave velocity (cfPWV; two-site tonometry with SphygmoCor) was used as a reference. A brachial single-cuff oscillometric device assessed aortic pulse wave velocity (aoPWV; Arteriograph) for further comparisons. We extracted 136 established PPG waveform features and engineered 13 new with improved coupling to the finger blood pressure curve. Height-normalized pulse arrival time (NPAT) was derived using ECG. Machine learning methods were used to develop prediction models.

**Results:**

The best PPG-based models predicted cfPWV and aoPWV well (root-mean-square errors of 0.70 and 0.52 m/s, respectively), with minor improvements by adding NPAT. Repeatability and agreement were on par with the reference equipment. A new PPG feature, an amplitude ratio from the early phase of the waveform, was most important in modelling, showing strong correlations with cfPWV and aoPWV (*r* = −0.81 and −0.75, respectively, both *P *< 0.001).

**Conclusion:**

Using new features and machine learning methods, a brief finger PPG registration can estimate aortic stiffness without requiring additional information on age, anthropometry, or blood pressure. Repeatability and agreement were comparable to those obtained using non-invasive reference equipment. Provided further validation, this readily available simple method could improve cardiovascular risk evaluation, treatment, and prognosis.

## 1 Introduction

Arterial stiffness can be considered a summation of all key cardiovascular risk factors throughout the life of an individual. Increased arterial stiffness is a hallmark feature of vascular ageing and has several adverse consequences for the cardiovascular system (1, 2). Measures of large artery stiffness, particularly aortic stiffness, improve risk stratification beyond traditional risk scores and serve as independent markers for future cardiovascular events and mortality (3, 4).

Several methods exist to assess aortic stiffness. The measurement of the carotid–femoral pulse wave velocity (cfPWV) by tonometry at the carotid and femoral arteries is considered the best non-invasive method, while brachial single-cuff-based oscillometric methods, which primarily rely on pulse wave analysis, are easier to perform (5). However, these methods are seldom used outside research settings, mainly due to low availability and often cumbersome procedures. There is a need for new, convenient methods to facilitate the implementation of aortic stiffness measurements into clinical practice (6).

Photoplethysmography (PPG), widely available in pulse oximeters, measures light intensity changes in the skin and underlying tissue and vasculature; it could be a method to assess aortic stiffness. The PPG waveform contains valuable information about the cardiovascular system, including numerous features (i.e., characteristics) associated with vascular ageing (7, 8). The physiological interpretation of the PPG waveform is however not fully understood, and the repeatability and reproducibility among PPG waveform features are known to vary (8, 9). The finger PPG signal reflects the peripheral blood pressure (BP) signal but is also affected by, for instance, ambient temperature and vasoconstriction (10). Still, there may exist information in the BP signal that could be used to better understand the PPG signals.

The electrocardiogram (ECG) is another broadly available method that provides an electrical representation of the heart activity. ECG can also serve as a time reference, for instance, to calculate the travel time of a pulse from the heart to a peripheral sensor, i.e., the pulse arrival time (PAT). Finger PAT is associated with both PWV and BP and is used in many BP estimation devices (11).

Modern machine learning (ML) methods offer new possibilities to evaluate large biomedical datasets and develop prediction models. Given its tradition of analysing waveform features, the field of PPG is well suited for these methods (12), and some previous studies have used ML and finger PPG for the classification of high vs. low cfPWV (13) and wrist PPG for the estimation of cfPWV (14). Jang et al. also used finger PPG features in linear regression models to predict brachial–ankle PWV (15).

PPG sensors are common in healthcare settings but are also increasingly becoming available in wearable consumer devices, like smartwatches, fitness trackers, and smart rings, often complemented with single-lead ECG. The increasing accessibility and ease of use warrant further studies on best utilization of these signals for cardiovascular risk assessments (16).

We hypothesized that waveform features from a short registration of finger PPG, alone or in combination with single-lead ECG, could be used to assess aortic stiffness using ML methods. In addition, we aimed to develop improved PPG features that provide a more reliable reflection of aortic stiffness.

## 2 Materials and methods

### 2.1 Participants

We invited volunteers aged 20–75 years from among colleagues and students at Danderyd University Hospital, Stockholm (Sweden) to participate during 2020–2021, provided they did not have chronic atrial fibrillation or significant kidney disease (there were no other criteria for exclusion).

### 2.2 Study protocol

The weight of each subject was measured, and information on height, medications, tobacco use, and a standard medical history was collected. All participants were instructed to avoid caffeine, alcohol, tobacco, and large meals at least 4 h prior to the examinations and to take any medications as prescribed. During the first visit, the distances from the jugulum (suprasternal notch) to the right common carotid artery pulsating site and from the jugulum to the right femoral artery pulsating site were measured using inelastic tape and the distance from the jugulum to the symphysis was measured using a caliper.

After at least 10 min of rest in the supine position, BP measurements and assessments of PWV respectively PPG, ECG, and finger BP recordings were performed in random order as groups, as described in the following. The measurements were performed in a quiet, dimly lit room at an ambient temperature of 21–24°C and repeated within 2 weeks under similar conditions. The same physician (HH) performed all measurements in all participants.

### 2.3 Blood pressure and assessments of pulse wave velocity

As the reference method, the original SphygmoCor device (AtCor Medical Pty. Ltd., West Ride, Australia) was used with a tonometer (Millar Instruments, Houston, TX, USA) for sequential collection of the right carotid and right femoral pulse waves, which were gated with the ECG signal (17, 18). The pulse travel distance was calculated as the distance from the jugulum to the right femoral artery minus the distance from the jugulum to the right carotid artery, and the cfPWV was calculated by the device as this distance divided by the average pulse travel time (19). Two measurements were performed (a third was added if the first two measurements differed by more than 0.5 m/s), and the mean value of these was used as the cfPWV for the visit.

For complementary evaluation, the brachial single-cuff-based Arteriograph device (Tensiomed Ltd., Budapest, Hungary) was used, which utilizes the oscillometric method for BP measurements and an occlusion technique to obtain aortic pulse wave velocity (aoPWV) (20–22). Using the right arm, one standard BP measurement was taken, and subsequently two measurements [also giving standard BP, heart rate (HR), and mean arterial pressure through diastolic + (systolic—diastolic)/3] of aoPWV, which by the device is calculated from the jugulum-to-symphysis distance and back divided by the return time of the reflected pulse wave. The mean values of the last two BP measurements and the two aoPWV recordings were used as the supine resting BP and aoPWV for the visit. The Arteriograph measurements were always performed before the SphygmoCor measurements.

On a population level, age and BP can be used to provide an estimated PWV (ePWV) (23, 24). For further evaluation of the current study results, ePWV was calculated by the equation for the normal population without cardiovascular risk factors using mean BP according to diastolic BP + 0.4 (systolic BP − diastolic BP) (23, 24).

### 2.4 Photoplethysmography, ECG, and finger blood pressure

An infrared (950 nm) reflectance PPG sensor clip (ADInstruments, Dunedin, New Zealand) was placed on the right index finger pulp, and three ECG electrodes were placed on the upper body to obtain a single-lead ECG (lead I). Continuous BP measurements using the volume-clamp method (Ohmeda 2300, Finapres, Englewood, CO, USA) were acquired from the right middle finger. All signals were integrated simultaneously via PowerLab (ADInstruments, Dunedin, New Zealand) at 1,000 Hz. Data were recorded during 6–7 min of supine rest.

### 2.5 Signal analysis and feature extraction

#### 2.5.1 Signal processing

A 20-s sequence was used, where all consecutive beats were of good quality, as defined by Orphanidou et al. (25), at a pulse rate variability between 5% and 10%, indicating a stable subject. This sequence length was a compromise between allowing for natural respiratory variations and avoiding artefacts. The signals were processed following procedures described by Elgendi (26) and Mejía-Mejía et al. (27), which included bandpass filtering (0.35–20 Hz), beat identification by the onset of the systolic upslope for the PPG and BP signals and by the R-wave for the ECG, beat length normalization, and averaging.

#### 2.5.2 Extraction of established features

**Figure 1:**
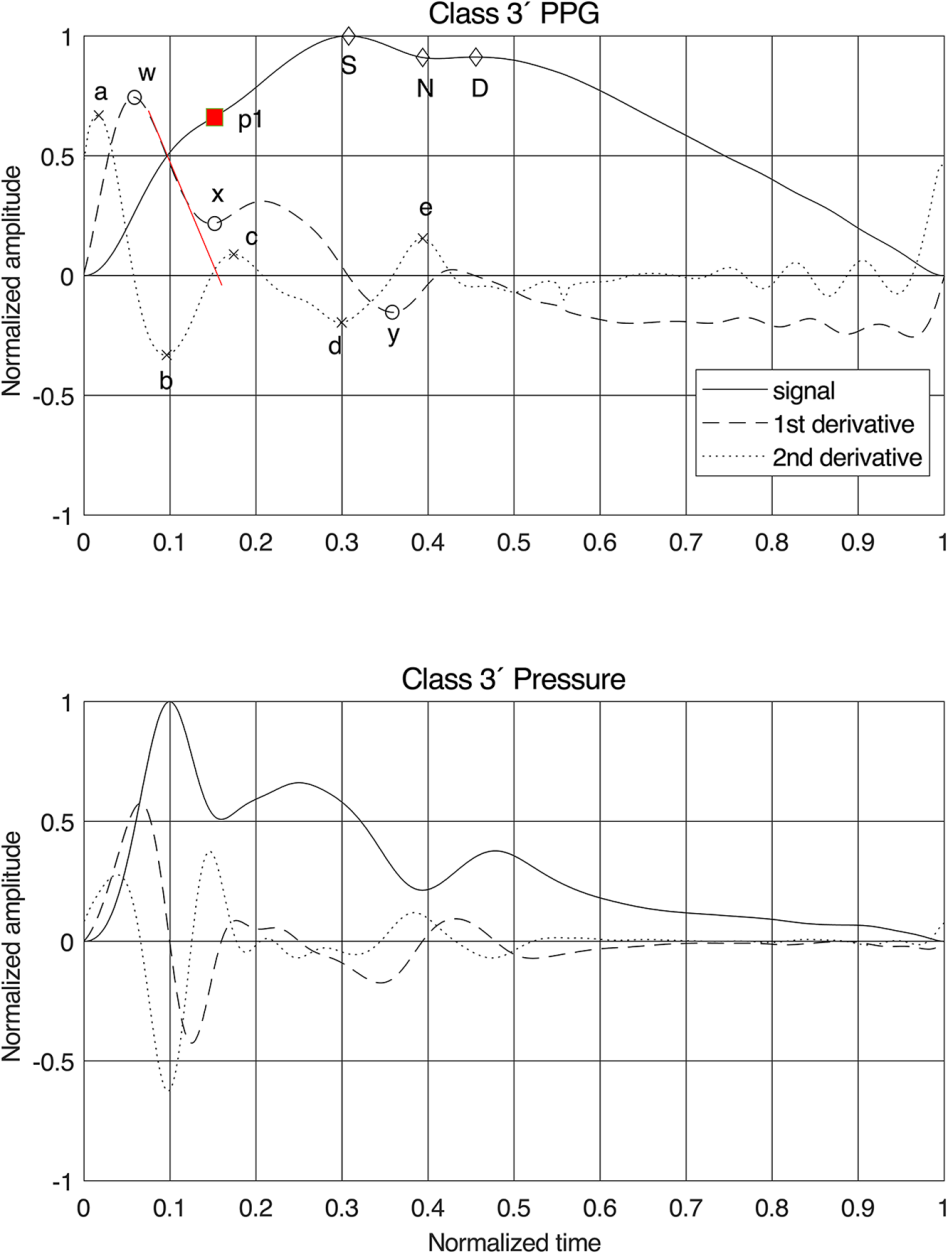
Normalized finger PPG and finger blood pressure signals with their first and second derivatives for class 3′. Fiducial points are given for the PPG signals and our proposal for the position of the first systolic peak “p1”. The red line on the first derivative is the tangent used for estimating the time stamp of “p1”. For PPG features, see Supplementary Table S1 for details.

**Figure 2:**
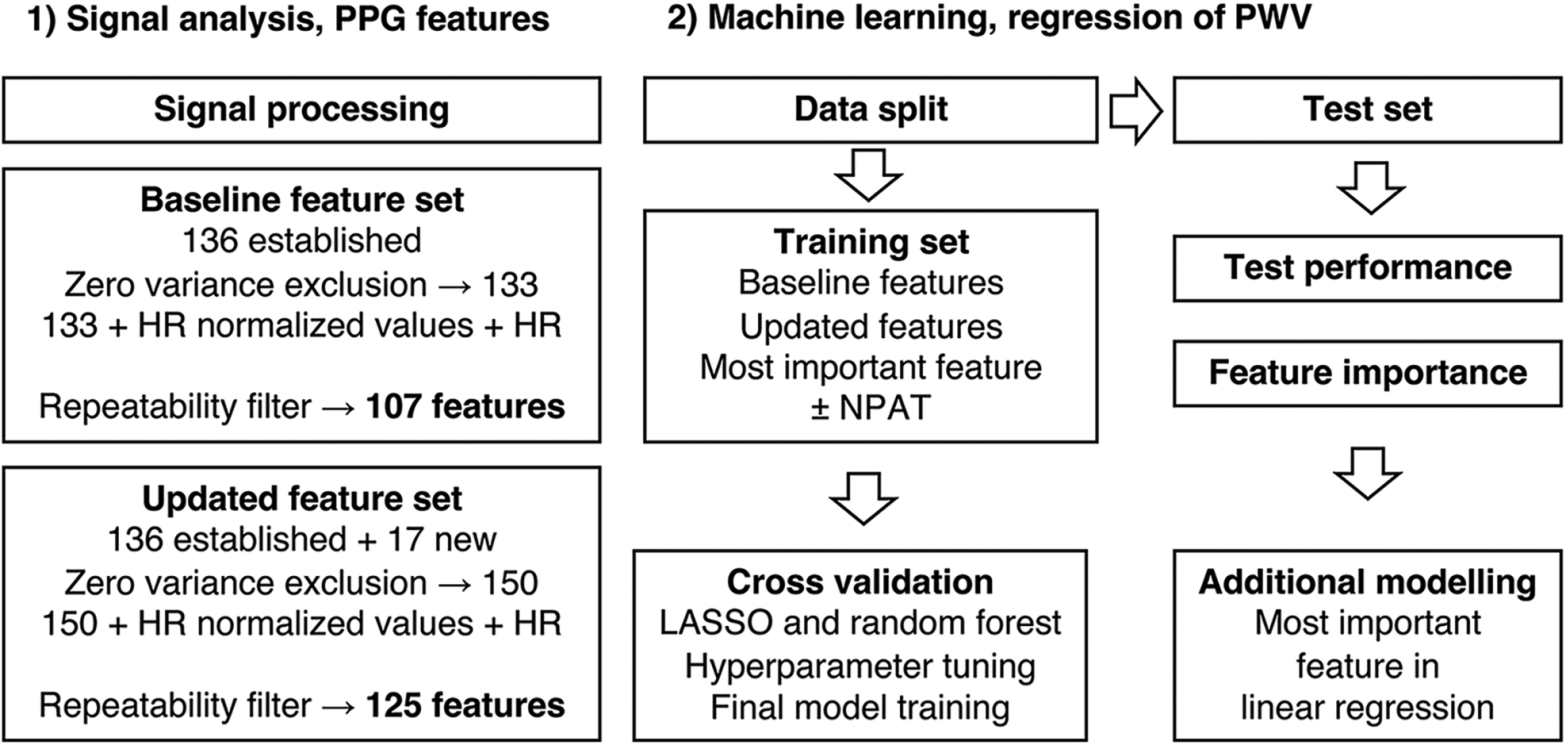
Signal analysis, feature sets, and machine learning.

The present state-of-the-art PPG fiducial points, as described by Elgendi (26) and Charlton et al. (7), were identified (Figure 1). The process was automated but with added manual supervision and generated 136 established features that were used in the baseline feature set (Figure 2).

#### 2.5.3 Understanding the pulse wave morphology

The local pressure in an artery can be decomposed into incident (from the cardiac systolic ejection) and reflected (mainly from vascular bifurcations) travelling waves whose phase speed is the local pulse wave velocity. At a peripheral measurement site such as the finger, the wave impedance is close to the characteristic impedance (28); that is, the reflection coefficient is low. Hence, the signal comprises a succession of “incident waves” arriving at time delays given by their respective travel paths and PWVs throughout the circulation.

#### 2.5.4 Interpretation of the photoplethysmogram

**Figure 3:**
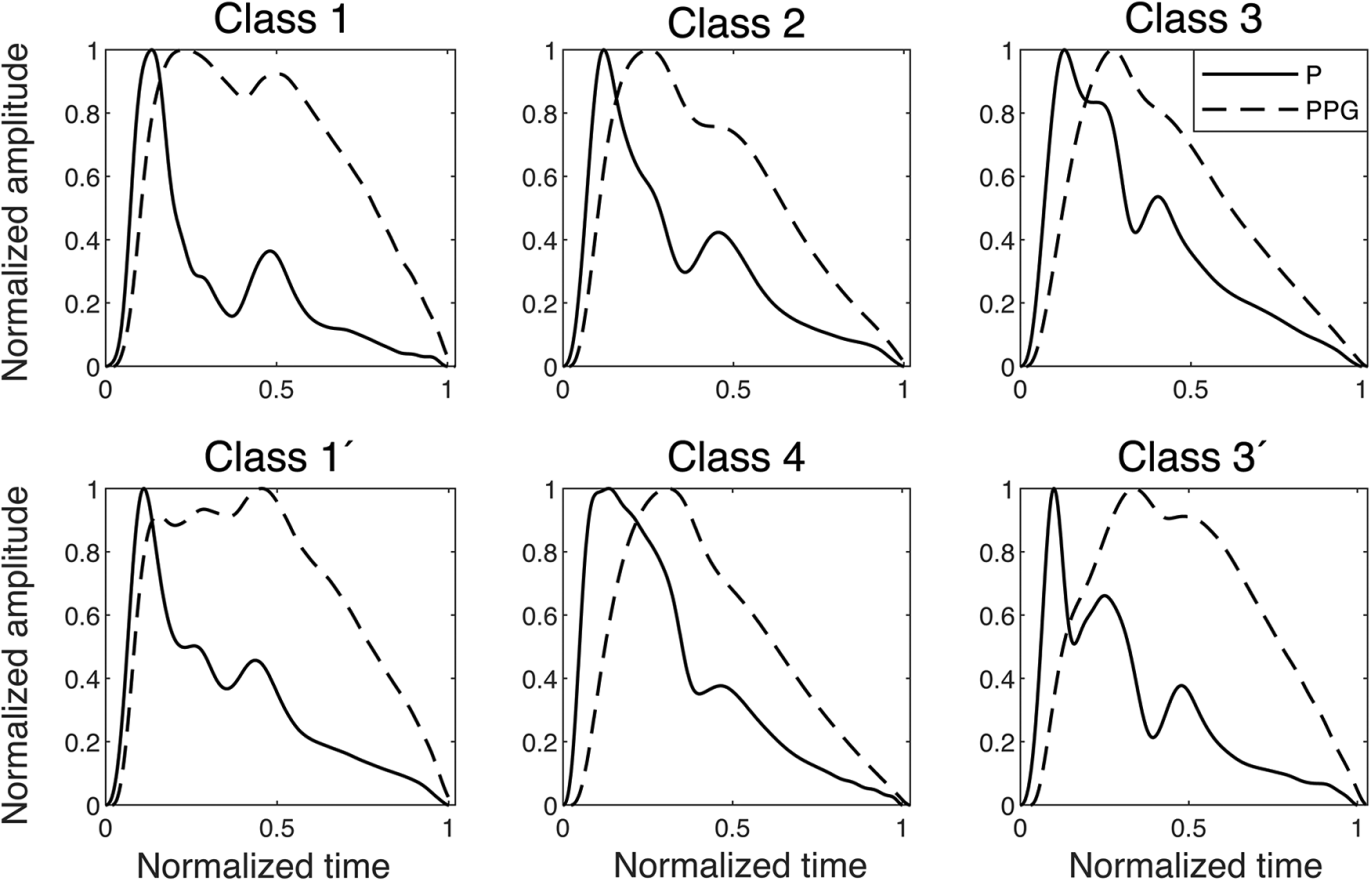
Examples of finger blood pressure and finger PPG signals of different classes found in the dataset. The signals are normalized, and the PPG signals are plotted with their relative time delay to the pressure signal, which in the onset is between 20 and 30 ms in all classes. P, pressure.

The PPG signal is merely a proxy for the actual BP/volume velocity of interest that lacks explainable transfer functions and also varies significantly between sites (29). Still, to evaluate the relationship between PPG and pressure, the participants were divided into four PPG waveform classes (10). Subgroups identified repeatedly for Class 1 and Class 3 were labelled with a prime (′). Class 1′ typically represented young, tall participants, and the common Class 3′, by Millasseau et al. (10), denoted “3bis,” is characterized by an anacrotic notch (an inflection point on the systolic upslope). The PPG classes were compared to simultaneous finger BP signals (Figure 3). An observation was the “inertia” in the PPG signal, where filling and emptying of the vascular beds had a different dynamic behaviour from the arterial pressure wave dynamics, which obscure details in the PPG.

#### 2.5.5 Engineering new features

As expected, the fiducial points for the derivatives were less obscured by the variations between the classes than the signals themselves (Figure 1). The pressure signal behaved as a single linear oscillator up to and around the first peak, where the first derivative displayed a linear gradient with a zero crossing at the first peak in the signal. For the PPG signal, however, the first derivative initially demonstrated a linear downslope after its first peak, but the delay in the signal caused a shift in the zero-crossing.

Therefore, a new method for identifying the first peak, “p1,” in the PPG signal was developed: by drawing a tangent from the initial linear part of the downslope of the first derivative after the fiducial point “w.” The zero-crossing of this tangent indicates the time stamp of “p1.” For Classes 1 and 1′, this point often coincided with “S” and in other classes, sometimes, with the anacrotic notch, as previously proposed in procedures to identify early and late systole (7, 30). Fiducial point “x” was defined at this timestamp as well, while “y” was chosen at the first local minimum after “x.” This method proved to work for all participants and all six classes.

For the updated feature set (Figure 2), 17 new features were constructed, of which 13 used the new “p1” as fiducial point. Assuming that the best coupling of the PPG signal to the arterial wall mechanics is found in the early part of the signal, resembling a single oscillator, one would expect the best result using fiducial points “a,” “b,” “w,” “x,” and “p1” (Figure 1). All features were also grouped by type and phase in the pulse waveform (Supplementary Table S1).

### 2.6 Statistical analysis

Descriptive data are presented as mean values ± standard deviations, median values with interquartile ranges, and proportions, as appropriate, unless stated otherwise. A two-sided probability (*P*) < 0.05 was considered significant. Coefficients of variation (CV) and within-subject coefficients of variation (WSCV, by the root-mean-square error (RMSE) method) were calculated. Bivariate correlations were studied using Pearson correlation analysis. Reference equipment agreements and prediction model performance were evaluated using root-mean-square error (RMSE), coefficients of determination (*R*^2^), and Bland–Altman analysis.

### 2.7 Machine learning

#### 2.7.1 Pre-processing and feature sets

All values were averaged between the two visits to generate independent observations (for values missing for either visit, the existing value was used as the average). Features with zero variance were excluded, and all features were divided by HR to adjust for possible HR effects (15, 31) in relation to aortic stiffness and included in each feature set. To ensure feature quality in terms of repeatability, features were also filtered on WSCV < 20%, which represented approximately the tertile with the best repeatability for each feature set. Extreme outliers were defined as values 3 ×  interquartile range greater than the third quartile or 3 × interquartile range less than the first quartile. All features were also centered and scaled. An overview of feature sets and machine learning methods is presented in Figure 2.

#### 2.7.2 Data split, resampling, and algorithms

The dataset was randomly split using 80% of the participants in the training set and 20% of the participants in the testing set. Due to the small sample size, we used 10-fold cross-validation, repeated 50 times, as the resampling method on the training set (32).

The established least absolute shrinkage and selection operator (LASSO) and random forest algorithms were used since they both have built-in variable importance scoring and variable selection and can handle multicollinearity, which is suitable for this high-dimensional dataset (33, 34). Simple and multiple linear regression were also performed with a subset of the features.

### 2.8 Software

The sensor signals were processed using MATLAB, version 2020b (The MathWorks Inc., MA, USA). Statistical analyses were performed using R software, version 4.3.1 (R Foundation for Statistical Computing, Vienna, Austria), and ML was conducted using the R packages glmnet, version 4.1.8, for modelling with LASSO, and ranger, version ranger 0.15.1, for modelling with random forest, both integrated into the R package tidymodels, version 1.1.0.

## 3 Results

### 3.1 Clinical characteristics

The clinical characteristics of the study participants are presented in Table 1. The participants represent a generally healthy population with normal values of aortic stiffness and no history of vascular disease. The body mass index ranged from 19 to 34 kg/m^2^.

**Table 1:** Clinical characteristics of the study cohort.

*n*	33
Age, years	44 (21–66)
Male sex	19 (58%)
Height, cm	176 ± 10
Weight, kg	78 ± 11
Body mass index, kg/m^2^	25.0 ± 3.6
History of hypertension	6 (18%)
History of vascular disease	0 (0%)
Previous smoking	5 (15%)
SBP, mmHg	119 ± 12
DBP, mmHg	71 ± 8
MAP, mmHg	87 ± 9
HR, bpm	60 ± 8
cfPWV, m/s	6.8 ± 1.1
aoPWV, m/s	7.6 ± 1.6

SBP, systolic blood pressure; DBP, diastolic blood pressure; MAP, mean arterial pressure.

Values are presented as mean ± standard deviation, median (range) or *n* (%). There were no current smokers. Blood pressures, HR, and pulse wave velocities are the mean values of visits 1 and 2 (SBP, DBP, MAP, HR, and aoPWV from the Arteriograph, and cfPWV using SphygmoCor).

### 3.2 Missingness, feature sets, and feature repeatability

There were seven PPG feature values and one aoPWV value missing (none for both visits for the same subject) where the existing value was used as the average. There were three PPG features with zero variance, which were excluded. After filtering on repeatability, the baseline feature set and the updated feature set consisted of 107 and 125 features, respectively. None of these included any extreme outliers.

**Figure 4:**
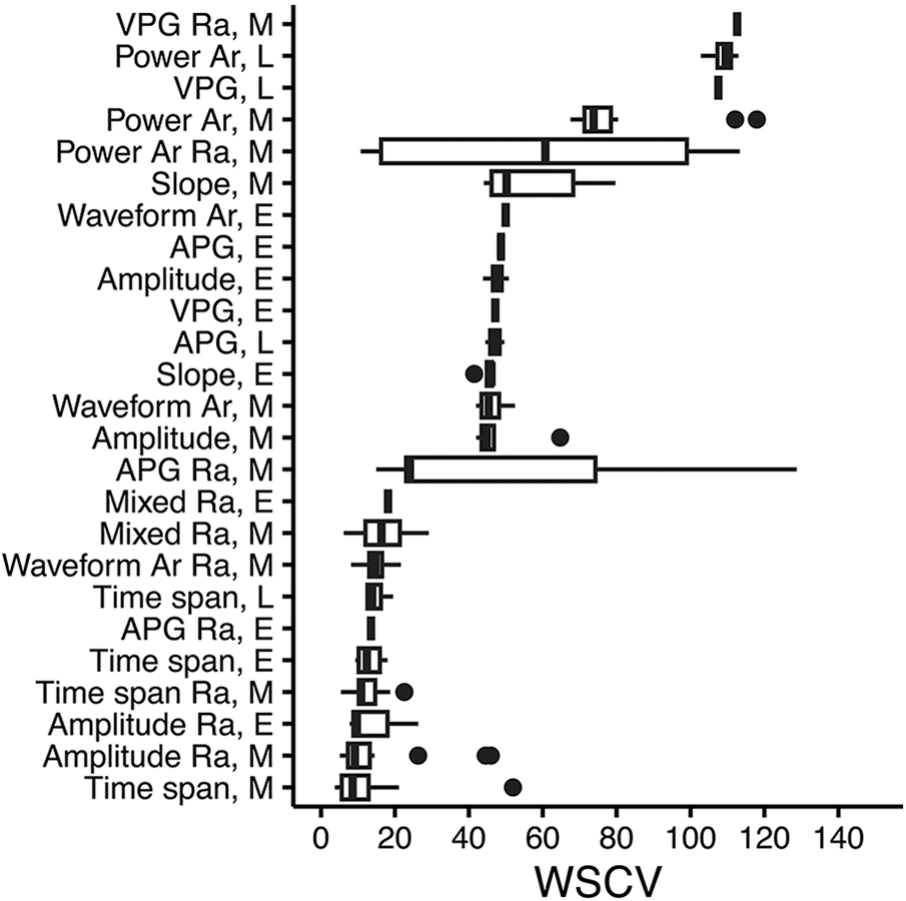
PPG feature type and phase in the waveform, according to repeatability (WSCV). Data were taken from the updated feature set. A total of 131 features with WSCV <150 are present in the figure. Definitions of phases are given in Supplementary Table S1. Box plots where vertical lines indicate the median, the lower and upper edges indicate the first and third quartiles, whiskers extend to the highest/lowest value within ±1.5 × interquartile range, and dots represent outliers. Ra, ratio; Ar, area; E, early; L, late; M, mixed; VPG, velocity plethysmogram; APG, acceleration plethysmogram.

A summary of all PPG-ECG features is presented in Supplementary Table S1. Feature types and feature phases according to repeatability (WSVC) are presented in Figure 4. Amplitude ratios, time spans, and time span ratios from the early or mixed phases had the lowest median WSCV (good repeatability).

### 3.3 Machine learning with the baseline feature set

When performing ML with the baseline feature set, the acceleration ratio “b/a” appeared as an important feature in all four ML cases but was considered most important only when using LASSO to predict cfPWV (Supplementary Figure S1). It showed moderate correlations with cfPWV and aoPWV (*r* = 0.54, *P *= 0.001 and *r* = 0.63, *P *< 0.001, respectively; Table 2). Feature “ms HR” was considered the most important when predicting aoPWV with both LASSO and random forest (Supplementary Figure S1), and it showed relatively strong correlations with cfPWV and aoPWV (*r* = −0.71 and *r* = −0.67, respectively, both *P *< 0.001; Table 2).

**Table 2:** Top PPG features and NPAT in relation to indices suggestive of vascular ageing.

	cfPWV		aoPWV		SBP		PP		Age	
	r	*P*	r	*P*	r	*P*	r	*P*	r	*P*
Tm ND HR	−0.63	<0.001	−0.51	0.003	−0.53	0.002	−0.30	0.088	−0.60	<0.001
b/a	0.54	0.001	0.63	<0.001	0.20	0.26	0.21	0.24	0.63	<0.001
Tm SD/Tm ss	−0.52	0.002	−0.49	0.004	−0.41	0.019	−0.06	0.74	−0.59	<0.001
Am b/Am S	−0.70	<0.001	−0.63	<0.001	−0.41	0.017	−0.20	0.27	−0.70	<0.001
Am b/Am S HR	−0.74	<0.001	−0.61	<0.001	−0.41	0.017	−0.21	0.24	−0.66	<0.001
ms	−0.54	0.001	−0.61	<0.001	−0.34	0.051	−0.21	0.24	−0.78	<0.001
ms HR	−0.71	<0.001	−0.67	<0.001	−0.41	0.018	−0.24	0.17	−0.81	<0.001
(b − e)/a	0.44	0.010	0.55	<0.001	0.21	0.25	0.28	0.11	0.44	0.010
Tm x/Tm ss	0.45	0.008	0.50	0.003	0.15	0.41	0.20	0.26	0.54	0.001
Am b/Am p1	−0.81	<0.001	−0.75	<0.001	−0.42	0.015	−0.29	0.10	−0.80	<0.001
Am b/Am p1 HR	−0.76	<0.001	−0.62	<0.001	−0.38	0.030	−0.25	0.16	−0.65	<0.001
Am c/Am p1	0.49	0.004	0.35	0.044	0.34	0.055	0.04	0.84	0.51	0.003
k v2	−0.59	<0.001	−0.57	<0.001	−0.32	0.072	−0.17	0.35	−0.62	<0.001
NPAT	−0.59	<0.001	−0.39	0.026	−0.61	<0.001	−0.40	0.021	−0.57	<0.001

SBP, systolic blood pressure; PP, pulse pressure; *P*, significance; HR, adjusted for heart rate.

Top PPG features from machine learning using the updated feature set, NPAT, and their linear correlations with indices suggestive of vascular ageing. The mean values of visits 1 and 2 were used. For PPG-ECG features, see Supplementary Table S1 for details.

Random forest had slightly better performance to predict both cfPWV and aoPWV, as compared to LASSO, in terms of test RMSE (Supplementary Table S2). For aoPWV, there were significantly higher RMSE values for training, as compared to testing. After removal of one extreme outlier for aoPWV (aoPWV = 13.0 m/s), resampling RMSE decreased, however test RMSE increased, compared to the initial modelling with all participants, which likely is due to overfitting (Supplementary Table S3).

Linear regression using only “b/a” exhibited good test performance for aoPWV but not for cfPWV (Supplementary Table S2).

### 3.4 Machine learning with the updated feature set

**Figure 5:**
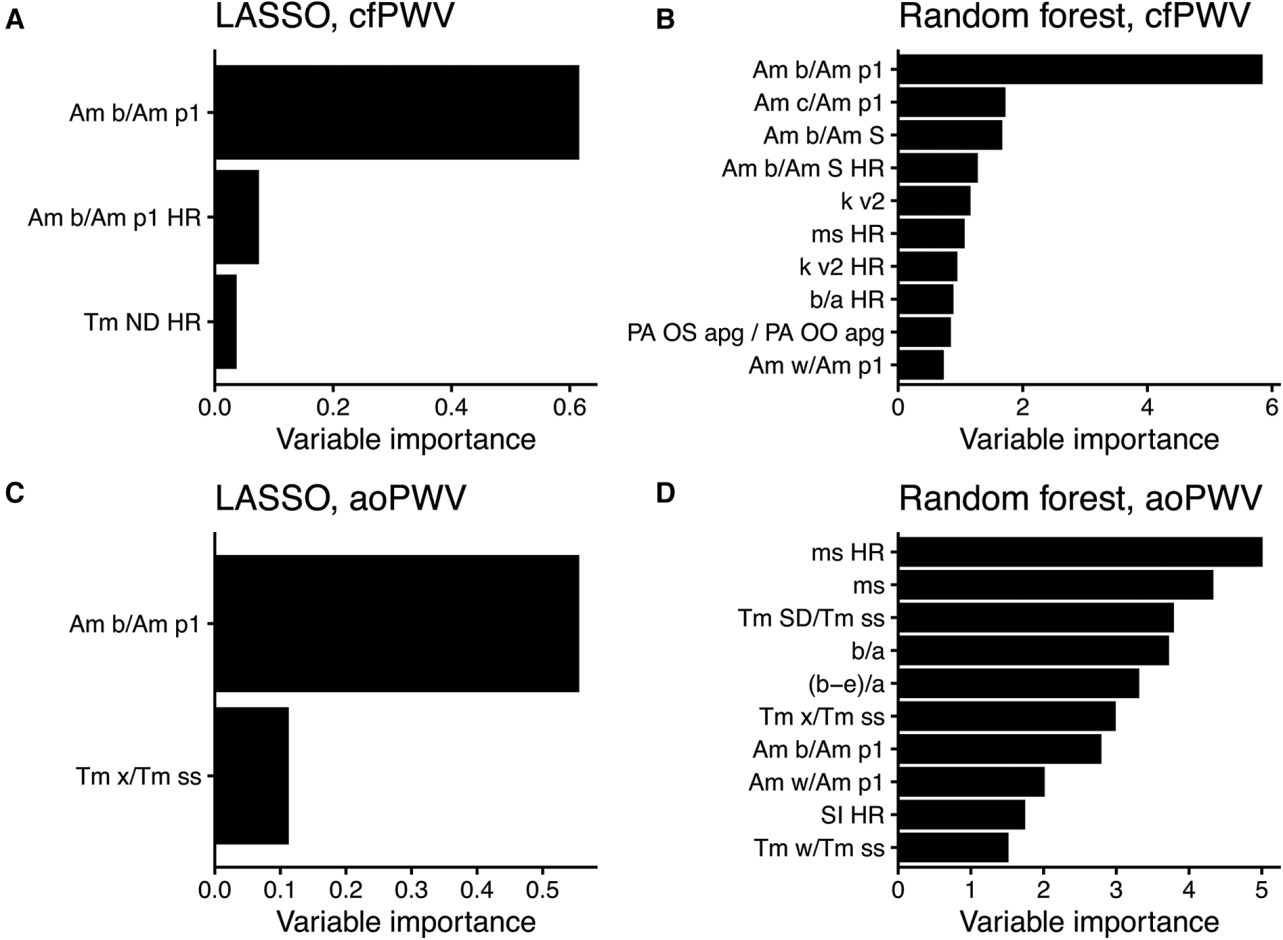
Variable importance scores from modelling with the updated feature set. (**A**) LASSO and (**B**) random forest to predict cfPWV, and (**C**) LASSO and (**D**) random forest to predict aoPWV. The scores use relative scales, and up to 10 features are shown per model. For PPG features, see Supplementary Table S1 for details. HR, adjusted for heart rate.

**Figure 6:**
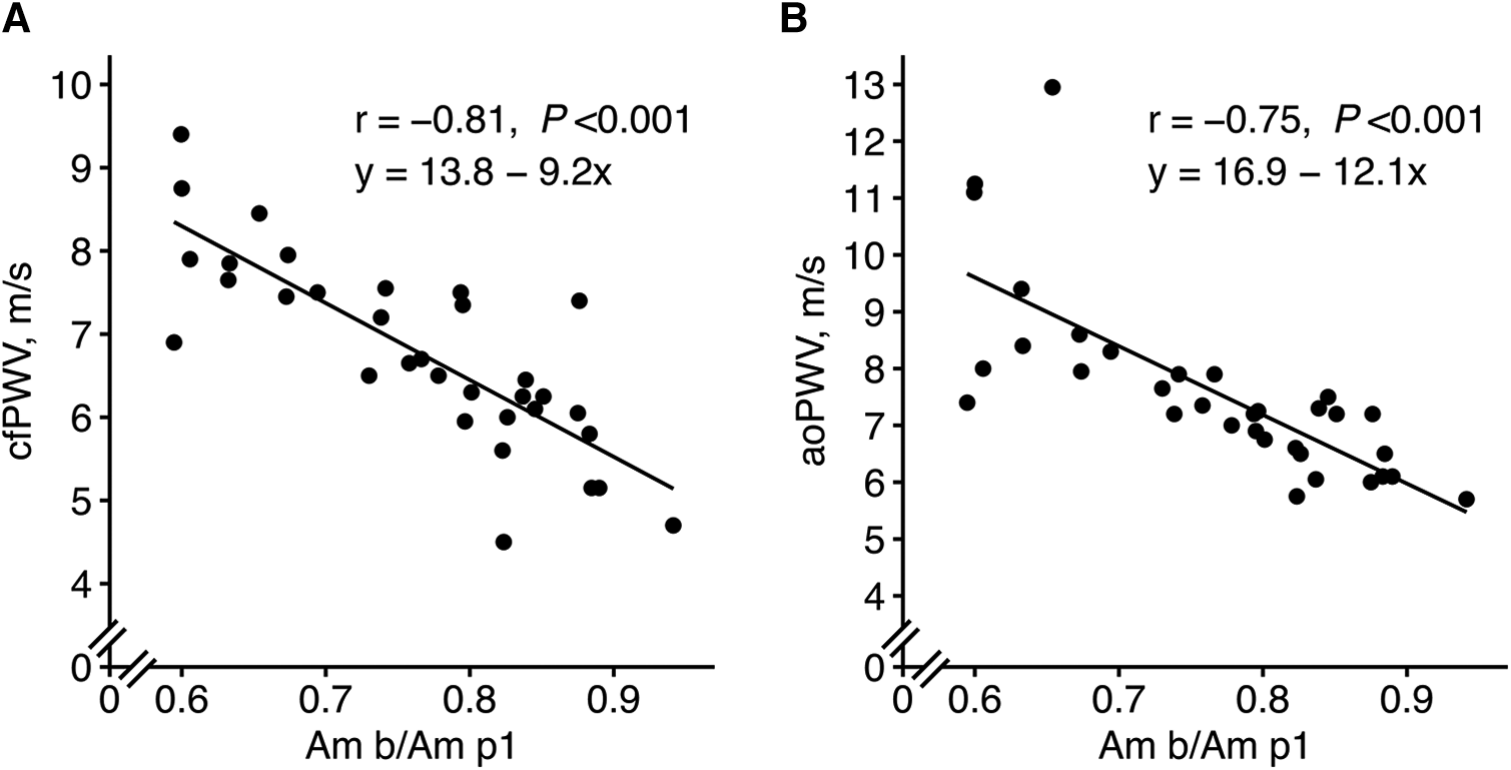
New PPG feature “Am b/Am p1” in relation to (**A**) cfPWV and (**B**) aoPWV. The mean values of visits 1 and 2 were used. Pearson correlation analysis with the r coefficient and *P*-value for each scatter plot. The lines represent simple linear regression lines. “Am b/Am p1”, a new amplitude ratio, see Supplementary Table S1 for details.

When performing ML with the updated feature set, the amplitude ratio “Am b/Am p1” appeared as the most important feature in both LASSO and random forest when predicting cfPWV and the most important for LASSO when predicting aoPWV (Figure 5). “Am b/Am p1” showed strong linear correlations with cfPWV and aoPWV (*r* = −0.81 and *r* = −0.75, respectively, both *P *< 0.001; Figure 6).

LASSO performed better than random forest in predicting cfPWV, but random forest was better at predicting aoPWV in terms of test RMSE (Table 3). Modelling with only “Am b/Am p1” in linear regression exhibited comparable test performance for cfPWV to the more complex models and showed the best test performance for aoPWV. Although the addition of the height-normalized PAT (NPAT) only marginally improved the test performance when added to “Am b/Am p1,” it did not improve other modelling cases (Table 3). The modelling performance between PAT and NPAT was compared using the updated feature set. NPAT showed slightly better performance for LASSO and linear regression in predicting cfPWV (data not shown), which is why NPAT was chosen instead of PAT.

**Table 3:** Performance of prediction models, based on the updated feature set.

Outcome	Algorithm	Features	Training, resampling performance		Testing, prediction performance	
			RMSE	*R* ^2^	RMSE	*R* ^2^
cfPWV	LASSO	Updated	0.69	0.87	0.70	0.74
cfPWV	LASSO	Updated + NPAT	0.66	0.86	0.70	0.72
cfPWV	Random forest	Updated	0.76	0.80	0.80	0.71
cfPWV	Random forest	Updated + NPAT	0.76	0.80	0.80	0.73
cfPWV	Linear regression	Am b/Am p1	0.60	0.91	0.76	0.71
cfPWV	Linear regression	Am b/Am p1 + NPAT	0.56	0.90	0.72	0.68
aoPWV	LASSO	Updated	1.16	0.84	0.76	0.92
aoPWV	LASSO	Updated + NPAT	1.16	0.84	0.76	0.92
aoPWV	Random forest	Updated	1.13	0.84	0.64	0.78
aoPWV	Random forest	Updated + NPAT	1.13	0.84	0.66	0.76
aoPWV	Linear regression	Am b/Am p1	1.00	0.90	0.52	0.92
aoPWV	Linear regression	Am b/Am p1 + NPAT	1.07	0.89	0.52	0.92

“Am b/Am p1”, a new amplitude ratio.

Prediction models for estimation of cfPWV or aoPWV using different machine learning algorithms and features. See Supplementary Table S1 for details on “Am b/Am p1” and NPAT.

**Figure 7:**
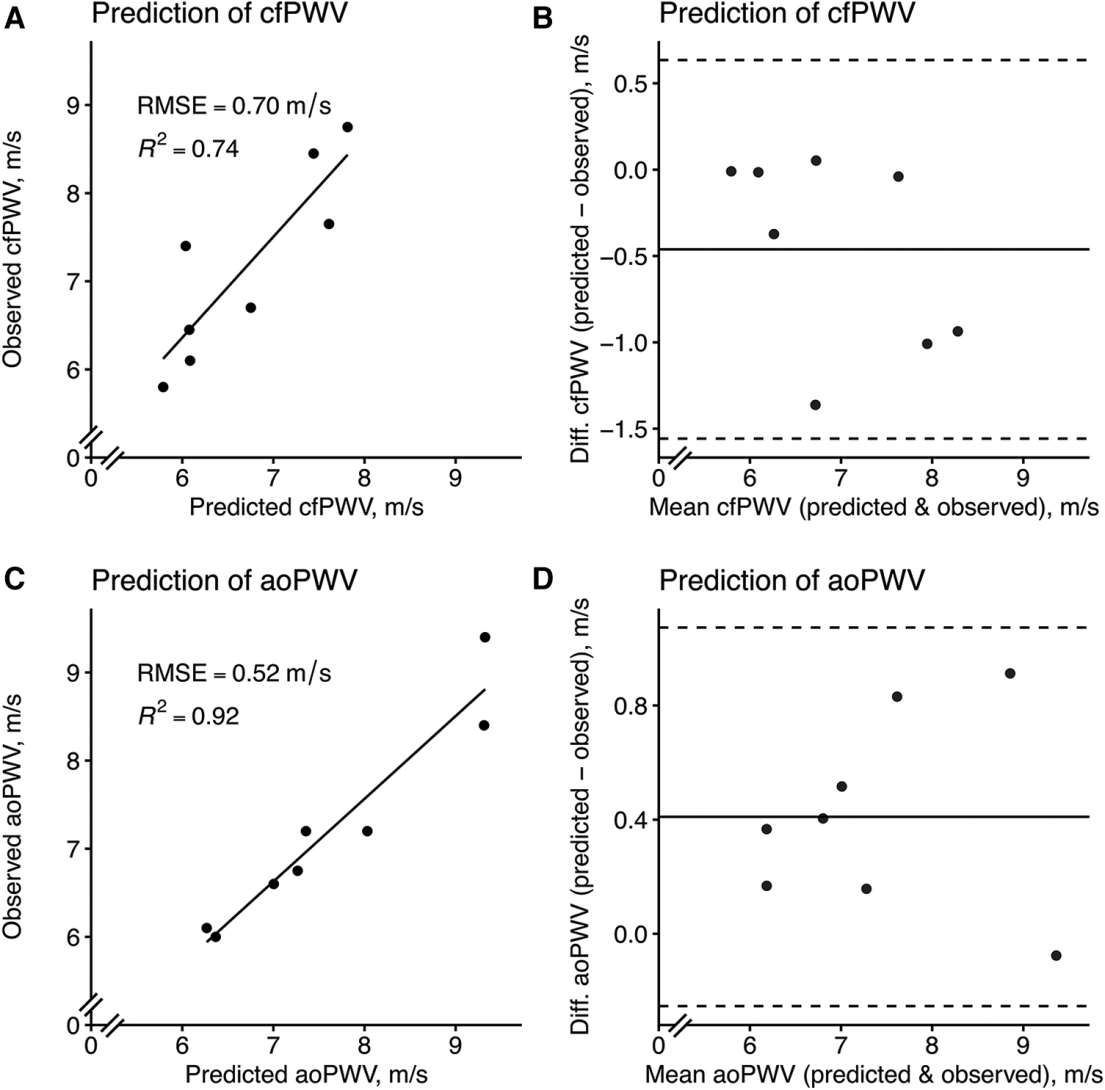
Model prediction of cfPWV and aoPWV in the test set using the best PPG-based models. (**A**) A LASSO model with the (**B**) corresponding Bland–Altman plot and (**C**) a linear regression model using only “Am b/Am p1” with the (**D**) corresponding Bland–Altman plot. For scatterplots, the solid line represents a simple linear regression line. For Bland–Altman plots, the solid line represents the bias (mean error) and the dashed lines represent the limits of agreement, which is ±1.96 times the standard deviation of the errors. “Am b/Am p1”, a new amplitude ratio, see Supplementary Table S1 for details.

The agreements of the best PPG-based prediction models, with the reference equipment, are found in Figure 7.

As when modelling aoPWV with the baseline feature set, there were also higher RMSE values for resampling as compared to testing (Table 3), and the behaviour with increased test RMSE was similar when removing the same extreme aoPWV outlier (Supplementary Table S4).

### 3.5 Top features, repeatability, and relations to indices suggestive of vascular ageing

The top five features from each modelling with LASSO and random forest using the updated feature set were aggregated, together with NPAT, and evaluated according to repeatability and associations with indices suggestive of vascular ageing.

In terms of repeatability, three out of four PPG features with the lowest WSCV were amplitude ratios derived from the early part of the PPG wave. NPAT had a lower WSCV compared to reference equipment, which in turn all had lower WSCV values than almost all PPG features (Table 4).

**Table 4:** Measures of dispersion and repeatability for top PPG features, NPAT, and reference equipment.

	Visit 1			Visit 2			Visit 1 vs. visit 2		
	Mean	SD	CV	Mean	SD	CV	r	*P*	WSCV
Tm ND HR	0.001	0.001	42.8	0.001	0.001	39.0	0.87	<0.001	17.2
b/a	−0.602	0.129	−21.4	−0.642	0.147	−23.0	0.71	<0.001	13.6
Tm SD/Tm ss	0.188	0.066	34.9	0.194	0.067	34.3	0.81	<0.001	13.6
Am b/Am S	0.682	0.174	25.5	0.679	0.165	24.2	0.77	<0.001	14.5
Am b/Am S HR	0.012	0.004	31.1	0.012	0.004	31.5	0.82	<0.001	14.8
ms	10.1	2.31	22.9	9.88	2.09	21.2	0.63	<0.001	14.0
ms HR	0.170	0.043	25.4	0.167	0.042	25.3	0.68	<0.001	14.3
(b − e)/a	−0.806	0.151	−18.7	−0.861	0.185	−21.5	0.53	0.001	15.0
Tm x/Tm ss	0.154	0.030	19.8	0.160	0.032	20.2	0.66	<0.001	10.9
Am b/Am p1	0.769	0.109	14.2	0.762	0.109	14.4	0.67	<0.001	9.72
Am b/Am p1 HR	0.013	0.003	21.9	0.013	0.003	22.3	0.83	<0.001	10.0
Am c/Am p1	1.02	0.077	7.58	1.01	0.097	9.51	0.55	<0.001	5.95
k v2	1.58	0.189	11.9	1.62	0.216	13.3	0.76	<0.001	6.15
NPAT	117	9.61	8.21	118	9.63	8.18	0.86	<0.001	3.06
cfPWV, m/s	6.74	1.17	17.3	6.80	1.18	17.4	0.88	<0.001	6.07
aoPWV, m/s	7.66	1.80	23.4	7.55	1.52	20.2	0.90	<0.001	5.80
SBP, mmHg	119	12.3	10.3	119	12.7	10.7	0.86	<0.001	3.79
DBP, mmHg	71.5	9.12	12.8	70.5	8.20	11.6	0.85	<0.001	4.72
HR, bpm	59.7	7.51	12.6	59.7	9.16	15.4	0.76	<0.001	6.46

*P*, significance; HR, adjusted for heart rate; SBP, systolic blood pressure; DBP, diastolic blood pressure.

Measures of dispersion and repeatability between visits 1 and 2 with values for top PPG features from machine learning using the updated feature set, NPAT, and measurements from reference equipment (cfPWV using SphygmoCor; aoPWV, SBP, DBP, and HR using Arteriograph). For PPG-ECG features, see Supplementary Table S1 for details.

In relation to different indices suggestive of vascular ageing, “Am b/Am p1” showed the highest associations with both cfPWV and aoPWV, NPAT with both systolic BP and pulse pressure, and “ms HR” with age (Table 2).

### 3.6 ePWV estimated by baseline risk factors to predict actual PWV

The calculated ePWV showed similar correlations with PWV (cfPWV *r* = 0.79, and aoPWV *r* = −0.71, both *P *< 0.001; Supplementary Figure S2) as the PPG feature “Am b/Am p1” (cfPWV *r* = −0.81, and aoPWV *r* = −0.75, both *P *< 0.001; Figure 6).

### 3.7 Reference equipment, repeatability, and agreement

WSCV values for reference equipment are listed in Table 4. To also illustrate repeatability, cfPWV and aoPWV were evaluated with coefficients of determination, which were similar between the two, and by Bland–Altman analysis, which was superior for cfPWV (Supplementary Figures S3A–D). The agreement between cfPWV and aoPWV showed a lower coefficient of determination and a Bland–Altman plot with more spread and four values outside the limits of agreement. cfPWV values were generally slightly lower than aoPWV values (Supplementary Figures S3E,F).

## 4 Discussion

This proof-of-concept study is the first to report on finger-PPG-based estimation of aortic stiffness against well-validated non-invasive methods while using improved features and ML methods. We provide four main findings. First, finger-PPG using advanced signal analysis and ML models can be used to estimate aortic stiffness without the need for additional information on age, anthropometry, or BP. Second, the ECG-based NPAT did not significantly improve model performance. Third, high feature quality can be ensured by improved fiducial point selection and assessing repeatability. Fourth, a new amplitude ratio “Am b/Am p1,” developed from an integral physiological understanding of the PPG wave, was identified as the most important feature for estimating aortic stiffness.

### 4.1 PPG-based prediction models

We developed several prediction models that predicted the two different estimates of aortic stiffness well. The best performance was obtained using the updated feature set, which utilized an improved identification of the first systolic peak. Simple models performed well; in fact, using only one of the new features in simple linear regression. LASSO and linear regression models are easily interpreted and shared with others, as encouraged for medical artificial intelligence and ML, although also more complex algorithms are prone to play a role in the future (12, 35). Our best models showed similar performance regarding repeatability and agreement to the reference equipment. Of note, both the SphygmoCor and Arteriograph devices have been validated invasively (18, 21) and can predict future cardiovascular events and mortality (3, 36, 37). This notwithstanding, our results highlight previously shown discrepancies between the methods, which have included both wide limits of agreement and weak correlation in their estimation of PWV (38, 39). This may be related to their different technical approaches.

It is not possible to directly compare our prediction model results with other studies since no other study has used the same setup with only the finger PPG waveform, real subjects, and prediction of PWV as continuous outcome variable. However, our best cfPWV model generated better results (e.g., RMSE 0.70 m/s) than other studies with more complex sensors and continuous cfPWV as an outcome. Thus, Tavallali et al. developed a prediction model from the carotid artery pressure waveform to predict cfPWV (RMSE 1.12 m/s), and Jin et al. predicted cfPWV from the radial pressure waveform with an RMSE of 1.82 m/s (40, 41).

The addition of the ECG-based NPAT did not significantly improve the performance of any of the models, indicating that the information regarding aortic stiffness is already sufficiently represented in the PPG signal. Thus, our results show that prediction models based on PPG alone can estimate aortic stiffness with good performance.

### 4.2 High-quality features

After a comprehensive study of the interaction between PPG and the simultaneous pressure curve, we constructed new features where the identification of the first systolic peak, “p1”, was enhanced. By using the new “p1”, features with better coupling to the BP curve, and likely also to arterial wall mechanics, were developed. To our knowledge, continuous peripheral BP has not provided guidance in previous studies regarding the development of PPG fiducial points, as in our study. Another way to ensure high feature quality was to filter features by WSCV, a measure of repeatability, before ML. There were large differences among the different feature types and waveform phases, with the best repeatability for amplitude ratios, time spans, and time span ratios from the early or mixed phases of the PPG waveform. Using only features with high repeatability should result in prediction models with improved performance in this aspect. Previous work studying repeatability also reported differences between features (31, 42, 43), but our study is more exhaustive and also investigates feature phases, which has not been published before. In summary, we ensured high feature quality by studying the BP curve and used only features with good repeatability for ML in a novel manner.

### 4.3 New essential amplitude ratio

ML identified the amplitude ratio “Am b/Am p1” as the most important feature. “Am b/Am p1” also showed strong linear correlations with both cfPWV and aoPWV (*r* = −0.81 and *r* = −0.75, respectively), which are stronger than what has been reported before; for the “spring constant” with cfPWV (*r* = −0.72) (44), the ageing index “(b − c − d − e)/a” with cfPWV (*r* = 0.65) (31), and for the stiffness index with central PWV (*r* = 0.65, *r* = 0.58 and *r* = 0.66) (45–47). Amplitudes “Am b” and “Am p1,” which together construct the ratio, are both located in the early phase of the PPG wave, and the ratio has good repeatability. “Am b” is defined as the PPG amplitude for acceleration “b,” and the “Am p1” is the amplitude of “p1,” the direct systolic peak, updated with our method for identification. The amplitude ratio decreases with higher PWV; that is, the amplitude, “Am p1,” becomes relatively larger and the amplitude before, “Am b,” becomes relatively smaller. Physiologically, this feature may represent a resistance in the aorta, which develops at the end of early systole. It is similar to the previously studied “spring constant” (44) and “slope of the rising front” (48), but these features did not perform equally well in our study. Of note, “Am b/Am p1” also showed stronger relations to central PWV compared to “b/a” (*r* = 0.54 to cfPWV, and *r* = 0.63 to aoPWV) and “ms HR” (*r* = −0.71 to cfPWV, and *r* = −0.67 to aoPWV)—features that were important using the baseline feature set. “Am b/Am p1” also showed the highest correlations with the reference equipment, which, although using different techniques, share much information.

Taken together, the new essential amplitude ratio “Am b/Am p1” was identified as a strong predictor of aortic stiffness that is also physiologically explainable. This should lead to more focus on this particular area of the PPG waveform when estimating aortic stiffness instead of indices that rely on, for instance, the timing between peaks “S” and “D,” like the stiffness index, or the uninterpretable ageing index “(b − c − d − e)/a,” or the “spring constant” that relies on the total systolic amplitude “Am S”, which does not correctly represent the true first systolic peak in all PPG classes.

### 4.4 Limitations

There are several shortcomings to this proof-of-concept study. First, results from studies based on a small sample size must always be interpreted with caution, and the small dataset prevented us from having an ideal modelling workflow, which should have included a training set, a validation set, and an external validation set. Second, the limited range for PWV in these generally healthy participants may affect the generalizability of the results. Third, there are potential confounding factors such as skin tone and obesity, which may influence PPG signals (49). However, utilizing finger PPG with a reflectance sensor on the finger pulp, the signal should be less influenced by skin tone since the skin in the palm and finger pulp generally contains little melanin. Obesity may affect the PPG signal due to differences in capillary density and skin thickness. However, obesity has not been shown to specifically affect the PPG signal using the finger pulp (49), and the participants in the current study were mostly of average weight. Fourth, the supervised process we used to identify fiducial points needs to be fully automated and programmed, which warrants further studies on its feasibility.

### 4.5 Clinical significance

Aortic stiffness is an important marker of cardiovascular risk but is seldom used in clinical practice as it is considered cumbersome to assess and requires specialized and expensive equipment. A simple method based on PPG could bridge several of these gaps due to low cost, high availability, and ease of use. This could also facilitate future studies on clinical applications of aortic stiffness in existing healthcare settings, but also using consumer devices like smartwatches, fitness trackers, and smart rings. Whether improved PPG-based estimates of aortic stiffness add clinically relevant independent information regarding future cardiovascular outcomes remains however to be evaluated in future properly designed studies.

### 4.6 Conclusion

This proof-of-concept study presents an improved method to estimate aortic stiffness by finger PPG alone through enhanced features and ML methods. The results were on par with non-invasive, well-validated methods for PWV regarding repeatability and agreement. Assessing aortic stiffness with this simple PPG method, which is already present in various consumer wearables, may facilitate cardiovascular risk evaluation and expedite broader studies of the added clinical value of arterial stiffness assessments.

## Data Availability

The datasets presented in this article are not readily available because they are dependent on new ethical approval and data-sharing agreements. Requests to access the datasets should be directed to Henrik Hellqvist, henrik.hellqvist@ki.se.
