# Supplementary material for: Estimation of aortic stiffness by finger photoplethysmography using enhanced pulse wave analysis and machine learning
Source: Front Cardiovasc Med. 2024 Mar 11;11:1350726. doi: 10.3389/fcvm.2024.1350726 (PMC10961400; doi:10.3389/fcvm.2024.1350726)
Supplement: Supplementary file 1 [file Datasheet1.docx]

Supplementary Material

# Supplementary Tables and Figures

**1.1 Supplementary Tables**

**Table S1:** List of all PPG-ECG features.

| No. | Feature | Alt. name | Definition | Type | Phase | Note | Reference |
| --- | --- | --- | --- | --- | --- | --- | --- |
| 1 | Tm a |  | tm(a) - tm(O) | Time span | Early | K, E |  |
| 2 | Tm w |  | tm(w) - tm(O) | Time span | Early | K, E |  |
| 3 | Tm b |  | tm(b) - tm(O) | Time span | Early | K, E |  |
| 4 | Tm S | Crest time, risetime | tm(S) - tm(O) | Time span | Mixed | K, E | Alty et al 2007 (1) |
| 5 | Tm c |  | tm(c) - tm(O) | Time span | Mixed | K, E |  |
| 6 | Tm y |  | tm(y) - tm(O) | Time span | Mixed | K, E |  |
| 7 | Tm N | t_sys_ | tm(N) - tm(O) | Time span | Mixed | K, E | Cho et al 2018 (2) |
| 8 | Tm D |  | tm(D) - tm(O) | Time span | Mixed | K, E |  |
| 9 | Tm ss |  | tm(next w) - tm(w) | Time span | Mixed | K, E |  |
| 10 | Tm Sc |  | tm(c) - tm(S) | Time span | Late | K, E |  |
| 11 | Tm Sd |  | tm(d) - tm(S) | Time span | Late | K, E |  |
| 12 | Tm Se |  | tm(e) - tm(S) | Time span | Late | K, E |  |
| 13 | Tm SD | PPT, ΔT | tm(D) - tm(S) | Time span | Late | K, E | Chowienczyk et al 1999 (3) |
| 14 | Tm ND |  | tm(D) - tm(N) | Time span | Late | K, E |  |
| 15 | Tm aa2 |  | tm(next a) - tm(a) | Time span | Mixed | K, E |  |
| 16 | Tm bc | t_b-c_ | tm(c) - tm(b) | Time span | Mixed | K, E | Ahn 2017 (4) |
| 17 | Tm bd | t_b-d_ | tm(d) - tm(b) | Time span | Mixed | K, E | Ahn 2017 (4) |
| 18 | Tm wb |  | tm(b) - tm(w) | Time span | Early | K, E |  |
| 19 | Tm wS |  | tm(S) - tm(w) | Time span | Mixed | K, E |  |
| 20 | Tm wc |  | tm(c) - tm(w) | Time span | Mixed | K, E |  |
| 21 | Tm wd |  | tm(d) - tm(w) | Time span | Mixed | K, E |  |
| 22 | Tm wz |  | tm(z) - tm(w) | Time span | Mixed | K, E |  |
| 23 | Tm ac |  | tm(c) - tm(a) | Time span | Mixed | K, E |  |
| 24 | Tm ab |  | tm(b) - tm(a) | Time span | Early | K, E |  |
| 25 | Am S |  | am(S) - am(O) | Amplitude | Mixed | K, E |  |
| 26 | Am a |  | am(a) - am(O) | Amplitude | Early | K, E |  |
| 27 | Am w |  | am(w) - am(O) | Amplitude | Early | K, E |  |
| 28 | Am b |  | am(b) - am(O) | Amplitude | Early | K, E |  |
| 29 | Am c |  | am(c) - am(O) | Amplitude | Mixed | K, E |  |
| 30 | Am y |  | am(y) - am(O) | Amplitude | Mixed | K, E |  |
| 31 | Am O2 |  | am(next O) - am(O) | Amplitude | Mixed | K, E |  |
| 32 | Am D |  | am(D) - am(O) | Amplitude | Mixed | K, E |  |
| 33 | Am N |  | am(N) - am(O) | Amplitude | Mixed | K, E |  |
| 34 | Am NS |  | am(S) - am(N) | Amplitude | Mixed | K, E |  |
| 35 | Am N/Am S |  | (am(N) - am(O)) / Am S | Amplitude ratio | Mixed | K, E |  |
| 36 | Am D/Am S |  | (am(D) - am(O)) / Am S | Amplitude ratio | Mixed | K, E |  |
| 37 | Am NS/Am S |  | (am(S) - am(N)) / Am S | Amplitude ratio | Mixed | K, E |  |
| 38 | Am DS/Am S | AI | (am(S) - am(D)) / Am S | Amplitude ratio | Mixed | K, E |  |
| 39 | w |  | vpg(am(w)) | VPG | Early | K, E |  |
| 40 | y |  | vpg(am(y)) | VPG | Late | K, E |  |
| 41 | z |  | vpg(am(z)) | VPG | Late | K, E |  |
| 42 | a |  | apg(am(a)) | APG | Early | K, E |  |
| 43 | b |  | apg(am(b)) | APG | Early | K, E |  |
| 44 | c |  | apg(am(c)) | APG | Late | K, E |  |
| 45 | d |  | apg(am(d)) | APG | Late | K, E |  |
| 46 | e |  | apg(am(e)) | APG | Late | K, E |  |
| 47 | cc |  | vpg(am(c)) | VPG | Late | K, E |  |
| 48 | dd |  | vpg(am(d)) | VPG | Late | K, E |  |
| 49 | z/w |  | z / w | VPG ratio | Mixed | K, E |  |
| 50 | y/w |  | y / w | VPG ratio | Mixed | K, E |  |
| 51 | cc/w |  | cc / w | VPG ratio | Mixed | K, E |  |
| 52 | dd/w |  | dd / w | VPG ratio | Mixed | K, E |  |
| 53 | b/a |  | b / a | APG ratio | Early | K, E | Takazawa et al 1998 (5) |
| 54 | c/a |  | c / a | APG ratio | Mixed | K, E | Takazawa et al 1998 (5) |
| 55 | d/a |  | d / a | APG ratio | Mixed | K, E | Takazawa et al 1998 (5) |
| 56 | e/a |  | e / a | APG ratio | Mixed | K, E | Takazawa et al 1998 (5) |
| 57 | (b–c–d–e)/a | AGI | (b–c–d–e) / a | APG ratio | Mixed | K, E | Takazawa et al 1998 (5) |
| 58 | (b–c–d)/a | AGI_mod_ | (b–c–d) / a | APG ratio | Mixed | K, E | Ushiroyama et al 2005 (6) |
| 59 | Ar OO |  | ar(OO) | Waveform area | Mixed | K, E |  |
| 60 | Ar OS |  | ar(OS) | Waveform area | Mixed | K, E |  |
| 61 | Ar Oc |  | ar(Oc) | Waveform area | Mixed | K, E |  |
| 62 | Ar ON | A1 | ar(ON) | Waveform area | Mixed | K, E |  |
| 63 | PA OS ppg |  | pa(OS) ppg | Power area | Mixed | K, E |  |
| 64 | PA wS ppg |  | pa(wS) ppg | Power area | Mixed | K, E |  |
| 65 | PA Sc ppg |  | pa(Sc) ppg | Power area | Mixed | K, E |  |
| 66 | PA Sd ppg |  | pa(Sd) ppg | Power area | Mixed | K, E |  |
| 67 | PA OS vpg |  | pa(OS) vpg | Power area | Mixed | K, E |  |
| 68 | PA wS vpg |  | pa(wS) vpg | Power area | Mixed | K, E |  |
| 69 | PA Sc vpg |  | pa(Sc) vpg | Power area | Late | K, E |  |
| 70 | PA Sd vpg |  | pa(Sd) vpg | Power area | Late | K, E |  |
| 71 | PA OS apg |  | pa(OS)apg | Power area | Mixed | K, E |  |
| 72 | PA wS apg |  | pa(wS) apg | Power area | Mixed | K, E |  |
| 73 | PA Sc apg |  | pa(Sc) apg | Power area | Late | K, E |  |
| 74 | PA Sd apg |  | pa(Sd) apg | Power area | Late | K, E |  |
| 75 | PA OO ppg |  | pa(OO) ppg | Power area | Mixed | K, E |  |
| 76 | PA OO vpg |  | pa(OO) vpg | Power area | Mixed | K, E |  |
| 77 | PA OO apg |  | pa(OO) apg | Power area | Mixed | K, E |  |
| 78 | Tm a/Tm ss |  | Tm a / Tm ss | Time span ratio | Mixed | K, E |  |
| 79 | Tm w/Tm ss |  | Tm w / Tm ss | Time span ratio | Mixed | K, E |  |
| 80 | Tm b/Tm ss |  | Tm b / Tm ss | Time span ratio | Mixed | K, E |  |
| 81 | Tm S/Tm ss | CTR, prop_s_ | Tm S / Tm ss | Time span ratio | Mixed | K, E | Wu et al 2010 (7) |
| 82 | Tm c/Tm ss |  | Tm c / Tm ss | Time span ratio | Mixed | K, E |  |
| 83 | Tm y/Tm ss |  | Tm y / Tm ss | Time span ratio | Mixed | K, E |  |
| 84 | Tm N/Tm ss |  | Tm N / Tm ss | Time span ratio | Mixed | K, E |  |
| 85 | Tm wz/Tm ss |  | Tm wz / Tm ss | Time span ratio | Mixed | K, E |  |
| 86 | Tm SD/Tm ss | prop_ΔT_ | Tm SD / Tm ss | Time span ratio | Mixed | K, E | Ahn 2017 (4) |
| 87 | Tm bb2/Tm ss |  | Tm bb2 / Tm ss | Time span ratio | Mixed | K, E |  |
| 88 | Am a/Am S |  | Am a / Am S | Amplitude ratio | Mixed | K, E |  |
| 89 | Am w/Am S | slope of the rising front | Am w / Am S | Amplitude ratio | Mixed | K, E | Pilt et al 2014 (8) |
| 90 | Am b/Am S |  | Am b / Am S | Amplitude ratio | Mixed | K, E |  |
| 91 | Am c/Am S |  | Am c / Am S | Amplitude ratio | Mixed | K, E |  |
| 92 | Am y/Am S |  | Am y / Am S | Amplitude ratio | Mixed | K, E |  |
| 93 | Am O2/Am S |  | Am O2 / Am S | Amplitude ratio | Mixed | K, E |  |
| 94 | IPA |  | ar(NO2) / ar(ON) | Waveform area ratio | Mixed | K, E |  |
| 95 | PIR |  | am(S) / am(O) | Amplitude ratio | Mixed | K, E |  |
| 96 | Ar OS/Ar OO |  | Ar OS/Ar OO | Waveform area ratio | Mixed | K, E |  |
| 97 | Ar Oc/Ar OO |  | Ar Oc/Ar OO | Waveform area ratio | Mixed | K, E |  |
| 98 | Ar ON/Ar OO |  | Ar ON/Ar OO | Waveform area ratio | Mixed | K, E |  |
| 99 | PA OS ppg / PA OO ppg |  | PA OS ppg / PA OO ppg | Power area ratio | Mixed | K, E |  |
| 100 | PA wS ppg / PA OO ppg |  | PA wS ppg / PA OO ppg | Power area ratio | Mixed | K, E |  |
| 101 | PA Sc ppg / PA OO ppg |  | PA Sc ppg / PA OO ppg | Power area ratio | Mixed | K, E |  |
| 102 | PA Sd ppg / PA OO ppg |  | PA Sd ppg / PA OO ppg | Power area ratio | Mixed | K, E |  |
| 103 | PA OS vpg / PA OO vpg |  | PA OS vpg / PA OO vpg | Power area ratio | Mixed | K, E |  |
| 104 | PA wS vpg / PA OO vpg |  | PA wS vpg / PA OO vpg | Power area ratio | Mixed | K, E |  |
| 105 | PA Sc vpg/ PA OO vpg |  | PA Sc vpg/ PA OO vpg | Power area ratio | Mixed | K, E |  |
| 106 | PA Sd vpg/ PA OO vpg |  | PA Sd vpg/ PA OO vpg | Power area ratio | Mixed | K, E |  |
| 107 | PA OS apg / PA OO apg |  | PA OS apg / PA OO apg | Power area ratio | Mixed | K, E |  |
| 108 | PA wS apg / PA OO apg |  | PA wS apg / PA OO apg | Power area ratio | Mixed | K, E |  |
| 109 | PA Sc apg/ PA OO apg |  | PA Sc apg/ PA OO apg | Power area ratio | Mixed | K, E |  |
| 110 | PA Sd apg/ PA OO apg |  | PA Sd apg/ PA OO apg | Power area ratio | Mixed | K, E |  |
| 111 | Sl Sc ppg |  | (am(c) - am(S)) / ((c - S)*st) | Slope | Late | K, E |  |
| 112 | Sl Sd ppg |  | (am(d) - am(S)) / ((d - S)*st) | Slope | Late | K, E |  |
| 113 | Sl bS ppg |  | (am(S) - am(b)) / ((S - b)*st) | Slope | Mixed | K, E |  |
| 114 | Sl bc ppg |  | (am(c) - am(b)) / ((c - b)*st) | Slope | Mixed | K, E |  |
| 115 | Sl bd ppg |  | (am(d) - am(b)) / ((d - b)*st) | Slope | Mixed | K, E |  |
| 116 | Sl wS ppg |  | (am(S) - am(w)) / ((S - w)*st) | Slope | Mixed | K, E |  |
| 117 | Sl OS ppg |  | (am(S) - am(O)) / ((S - O)*st) | Slope | Mixed | K, E |  |
| 118 | Sl ab ppg |  | (am(b) - am(a)) / ((b - a)*st) | Slope | Early | K, E |  |
| 119 | Sl ab apg |  | (apg(b) - apg(a)) / ((b - a)*st) | Slope | Early | K, E |  |
| 120 | Sl bS apg |  | (apg(S) - apg(b)) / ((S - b)*st) | Slope | Mixed | K, E |  |
| 121 | Sl bc apg | slope_b-c_ | (apg(c) - apg(b)) / ((c - b)*st) | Slope | Mixed | K, E | Ahn 2017 (4) |
| 122 | Sl bd apg | slope_b-d_ | (apg(d) - apg(b)) / ((d - b)*st) | Slope | Mixed | K, E | Ahn 2017 (4) |
| 123 | Sl be apg |  | (apg(e) - apg(b)) / ((e - b)*st) | Slope | Mixed | K, E |  |
| 124 | Sl Sc apg |  | (apg(c) - apg(S)) / ((c - S)*st) | Slope | Late | K, E |  |
| 125 | Sl wS apg |  | (apg(S) - apg(w)) / ((S - w)*st) | Slope | Mixed | K, E |  |
| 126 | Sl OS apg |  | (apg(S) - apg(O)) / ((S - O)*st) | Slope | Mixed | K, E |  |
| 127 | Tdia | t_dia_ | Tm aa2 - Tm N | Time span | Mixed | K | Ahn 2017 (4) |
| 128 | Tratio | t_ratio_ | Tm S / Tm N | Time span ratio | Mixed | K | Ahn 2017 (4) |
| 129 | IPR |  | 60 / Tm aa2 | Time span | Mixed | K | Lueken et al 2017 (9) |
| 130 | ms |  | w / Am S | Mixed ratio | Mixed | K | Alty et al 2003 (10) |
| 131 | RI |  | Am D / Am S | Amplitude ratio | Mixed | K | Chowienczyk et al 1999 (3) |
| 132 | (b-e)/a |  | (b-e)/a | APG ratio | Mixed | K | Baek et al 2007 (11) |
| 133 | IPAD |  | IPA + d/a | Mixed ratio | Mixed | K | Ahn 2017 (4) |
| 134 | SI | stiffness index | height / Tm SD | Time span | Late | K | Millasseau et al 2002 (12) |
| 135 | Tm p1p2 |  | tm(p2) - tm(p1) | Time span | Mixed | N |  |
| 136 | Tm p1 |  | tm(p1) - tm(O) | Time span | Early | N |  |
| 137 | Am p1 |  | am(O) - am(p1) | Amplitude | Early | N |  |
| 138 | Am N/Am p1 |  | (am(N) - am(O)) / Am p1 | Amplitude ratio | Mixed | N |  |
| 139 | Am D/Am p1 |  | (am(D) - am(O)) / Am p1 | Amplitude ratio | Mixed | N |  |
| 140 | Am NS/Am p1 |  | (am(S) - am(N)) / Am p1 | Amplitude ratio | Mixed | N |  |
| 141 | Am DS/Am p1 |  | (am(S) - am(D)) / Am p1 | Amplitude ratio | Mixed | N |  |
| 142 | Ar Op1 |  | ar(Op1) | Waveform area | Early | N |  |
| 143 | Tm x/Tm ss |  | tm(x) - tm(O) / Tm ss | Time span ratio | Mixed | N |  |
| 144 | Am a/Am p1 |  | Am a / Am p1 | Amplitude ratio | Early | N |  |
| 145 | Am w/Am p1 |  | Am w / Am p1 | Amplitude ratio | Early | N |  |
| 146 | Am b/Am p1 |  | Am b / Am p1 | Amplitude ratio | Early | N |  |
| 147 | Am c/Am p1 |  | Am c / Am p1 | Amplitude ratio | Mixed | N |  |
| 148 | Am y/Am p1 |  | Am y / Am p1 | Amplitude ratio | Mixed | N |  |
| 149 | Sl ax ppg |  | (am(x) - am(a)) / ((x - a)*st) | Slope | Early | N |  |
| 150 | Sl bx ppg |  | (am(x) - am(b)) / ((x - b)*st) | Slope | Early | N |  |
| 151 | k v1 | spring constant, *k* | apg(S) / (Am S - Am w) | Mixed ratio | Mixed | K | Wei 2013 (13) |
| 152 | k v2 |  | am(S) / (Am S - Am w) | Mixed ratio | Mixed | K | Charlton 2020 (14) |
| 153 | k v3 |  | apg(a) / (Am b - Am a) | Mixed ratio | Early | N |  |
| 154 | HR, bpm |  | 60 / tm(next O)-tm(O) | Time span |  |  |  |
| 155 | PAT |  | tm(O) - tm(ecg(R-peak)) | PPG-ECG feature |  |  |  |
| 156 | NPAT |  | PAT / height | PPG-ECG feature |  |  |  |

List of features with corresponding definitions, type, and for PPG, phase in the pulse waveform, where applicable. Alternative names from literature are listed. Previously known features are noted “K”. For most features, definitions and further details can be found in Elgendi (15), noted “E”. Features noted “N” are new and were engineered in the current study. Features previously associated with arterial stiffness have specific references added and most of these have been summarized by Charlton et al (16, 17). PPG features phases were defined as early if from, or dependent on features, from the onset “O”, in the origin, to “p1”; late, from, or dependent on features from after “p1” up until the next “O”, or mixed if stemming from both early and late phases (or from early phase but include features in the next beat). Many features are also presented in Figure 1. Tm/tm indicates time; Am/am, amplitude; VPG/vpg, velocity plethysmogram; APG/apg, acceleration plethysmogram; Ar/ar, area; PA/pa, power area; PPG/ppg, photoplethysmography; Sl, slope; st, sample time; HR, heart rate; PAT, pulse arrival time; NPAT, normalized PAT.

**References for Table S1**1. Alty SR, Angarita-Jaimes N, Millasseau SC, Chowienczyk PJ. Predicting arterial stiffness from the digital volume pulse waveform. IEEE Trans Biomed Eng. 2007;54(12):2268-75. doi: 10.1109/tbme.2007.897805

2. Cho SI, Negishi T, Tsuchiya M, Yasuda M, Yokoyama M. Estimation system of blood pressure variation with photoplethysmography signals using multiple regression analysis and neural network. International Journal of Fuzzy Logic and Intelligent Systems. 2018;18(4):229-36. doi: 10.5391/IJFIS.2018.18.4.229

3. Chowienczyk PJ, Kelly RP, MacCallum H, Millasseau SC, Andersson TL, Gosling RG, et al. Photoplethysmographic assessment of pulse wave reflection: blunted response to endothelium-dependent beta2-adrenergic vasodilation in type II diabetes mellitus. J Am Coll Cardiol. 1999;34(7):2007-14. doi: 10.1016/s0735-1097(99)00441-6

4. Ahn JM. New Aging Index Using Signal Features of Both Photoplethysmograms and Acceleration Plethysmograms. Healthcare Informatics Research. 2017;23(1):53-. doi: 10.4258/hir.2017.23.1.53

5. Takazawa K, Tanaka N, Fujita M, Matsuoka O, Saiki T, Aikawa M, et al. Assessment of vasoactive agents and vascular aging by the second derivative of photoplethysmogram waveform. Hypertension. 1998;32(2):365-70. doi: 10.1161/01.HYP.32.2.365

6. Ushiroyama T, Kajimoto Y, Sakuma K, Minoru U. Assessment of chilly sensation in Japanese women with Laser Doppler Fluxmetry and Acceleration Plethysmogram with Respect to Peripheral Circulation. Bull Osaka Med Coll 2005;51: 76–84. doi: 10.57371/00000405

7. Wu HT, Liu CC, Lin PH, Chung HM, Liu MC, Yip HK, et al. Novel application of parameters in waveform contour analysis for assessing arterial stiffness in aged and atherosclerotic subjects. Atherosclerosis. 2010;213(1):173-7. doi: 10.1016/j.atherosclerosis.2010.08.075

8. Pilt K, Meigas K, Kööts K, Viigimaa M. Photoplethysmographic signal rising front analysis for the discrimination of subjects with increased arterial ageing. Proc Estonian Acad Sci. 2014;63:309-14. doi: 10.3176/proc.2014.3.03

9. Lueken M, Feng X, Venema B, Misgeld BJE, Leonhardt S, editors. Photoplethysmography-based in-ear sensor system for identification of increased stress arousal in everyday life. 2017 IEEE 14th International Conference on Wearable and Implantable Body Sensor Networks (BSN); 2017 9-12 May 2017. doi: 10.1109/BSN.2017.7936013

10. Alty SR, Millasseau SC, Chowienczyc PJ, Jakobsson A, editors. Cardiovascular disease prediction using support vector machines. 2003 46th Midwest Symposium on Circuits and Systems; 2003 27-30 Dec. 2003. doi: 10.1109/MWSCAS.2003.1562297

11. Baek HJ, Kim JS, Kim YS, Lee HB, Park KS, editors. Second Derivative of Photoplethysmography for Estimating Vascular Aging. 2007 6th International Special Topic Conference on Information Technology Applications in Biomedicine; 2007 8-11 Nov. 2007. doi: 10.1109/ITAB.2007.4407346

12. Millasseau SC, Kelly RP, Ritter JM, Chowienczyk PJ. Determination of age-related increases in large artery stiffness by digital pulse contour analysis. Clin Sci (Lond). 2002;103(4):371-7. doi: 10.1042/cs1030371

13. Wei CC. Developing an effective arterial stiffness monitoring system using the spring constant method and photoplethysmography. IEEE Trans Biomed Eng. 2013;60(1):151-4. doi: 10.1109/TBME.2012.2207384

14. Charlton P. PulseAnalyse 2020 [PulseAnalyse is a tool for analysing arterial pulse waves.]. Available from: <https://github.com/peterhcharlton/pulse-analyse>. doi:

15. Elgendi M. PPG Signal Analysis. Boca Raton, FL: Taylor & Francis; 2020. doi: 10.1201/9780429449581

16. Charlton PH, Paliakaite B, Pilt K, Bachler M, Zanelli S, Kulin D, et al. Assessing hemodynamics from the photoplethysmogram to gain insights into vascular age: a review from VascAgeNet. Am J Physiol Heart Circ Physiol. 2022;322(4):H493-H522. doi: 10.1152/ajpheart.00392.2021

17. Charlton PH, Celka P, Farukh B, Chowienczyk P, Alastruey J. Assessing mental stress from the photoplethysmogram: a numerical study. Physiol Meas. 2018;39(5):054001. doi: 10.1088/1361-6579/aabe6a

Table S2: Performance of prediction models, based on the baseline feature set

|  | | | Training, resampling performance | | Testing, prediction performance | |
| --- | --- | --- | --- | --- | --- | --- |
| Outcome | Algorithm | Features | RMSE | *R*^2^ | RMSE | *R*^2^ |
| cfPWV | LASSO | Baseline | 0.77 | 0.79 | 0.84 | 0.61 |
| cfPWV | Random forest | Baseline | 0.80 | 0.78 | 0.72 | 0.77 |
| cfPWV | Linear regression | b/a | 0.89 | 0.82 | 1.04 | 0.31 |
| aoPWV | LASSO | Baseline | 1.26 | 0.77 | 0.87 | 0.65 |
| aoPWV | Random forest | Baseline | 1.14 | 0.84 | 0.70 | 0.66 |
| aoPWV | Linear regression | b/a | 1.28 | 0.87 | 0.79 | 0.78 |

Prediction models for estimation of cfPWV or aoPWV using different machine learning algorithms and features. RMSE indicates root mean square error; *R*^2^, coefficient of determination; cfPWV, carotid-femoral pulse wave velocity (using SphygmoCor); aoPWV, aortic pulse wave velocity (using Arteriograph); LASSO, least absolute shrinkage and selection operator; PPG, photoplethysmography; “b/a”, an established acceleration ratio, please see Supplementary Table S1 for definition.

Table S3: Performance of prediction models, based on the baseline feature set, after removal of one extreme outlier for aoPWV

|  | | | Training, resampling performance | | Testing, prediction performance | |
| --- | --- | --- | --- | --- | --- | --- |
| Outcome | Algorithm | Features | RMSE | *R*^2^ | RMSE | *R*^2^ |
| aoPWV | LASSO | Baseline | 0.97 | 0.83 | 1.00 | 0.53 |
| aoPWV | Random forest | Baseline | 1.04 | 0.81 | 1.01 | 0.45 |
| aoPWV | Linear regression | b/a | 1.05 | 0.82 | 0.77 | 0.61 |

Prediction models for estimation of aoPWV using different machine learning algorithms and features, after removal of one subject with an extreme outlier for aoPWV. RMSE indicates root mean square error; *R*^2^, coefficient of determination; cfPWV, carotid-femoral pulse wave velocity (using SphygmoCor); aoPWV, aortic pulse wave velocity (using Arteriograph); LASSO, least absolute shrinkage and selection operator; PPG, photoplethysmography; “b/a”, an established acceleration ratio; see Supplementary Table S1 for details.

Table S4: Performance of prediction models, based on the updated feature set, after removal of one extreme outlier for aoPWV

|  | | | Training, resampling performance | | Testing, prediction performance | |
| --- | --- | --- | --- | --- | --- | --- |
| Outcome | Algorithm | Features | RMSE | *R*^2^ | RMSE | *R*^2^ |
| aoPWV | LASSO | Updated | 0.86 | 0.88 | 0.78 | 0.76 |
| aoPWV | LASSO | Updated + NPAT | 0.86 | 0.88 | 0.78 | 0.76 |
| aoPWV | Random forest | Updated | 1.02 | 0.81 | 1.02 | 0.43 |
| aoPWV | Random forest | Updated + NPAT | 1.02 | 0.82 | 1.01 | 0.44 |
| aoPWV | Linear regression | Am b/Am p1 | 0.77 | 0.90 | 0.61 | 0.76 |
| aoPWV | Linear regression | Am b/Am p1 + NPAT | 0.80 | 0.89 | 0.54 | 0.84 |

Prediction models for estimation of aoPWV using different machine learning algorithms and features, after removal of one subject with an extreme outlier for aoPWV. RMSE indicates root mean square error; *R*^2^, coefficient of determination; cfPWV, carotid-femoral pulse wave velocity (using SphygmoCor); aoPWV, aortic pulse wave velocity (using Arteriograph); LASSO, least absolute shrinkage and selection operator; PPG, photoplethysmography; NPAT, normalized pulse arrival time; “Am b/Am p1”, a new amplitude ratio, see Supplementary Table S1 for details.

## Supplementary Figures

**
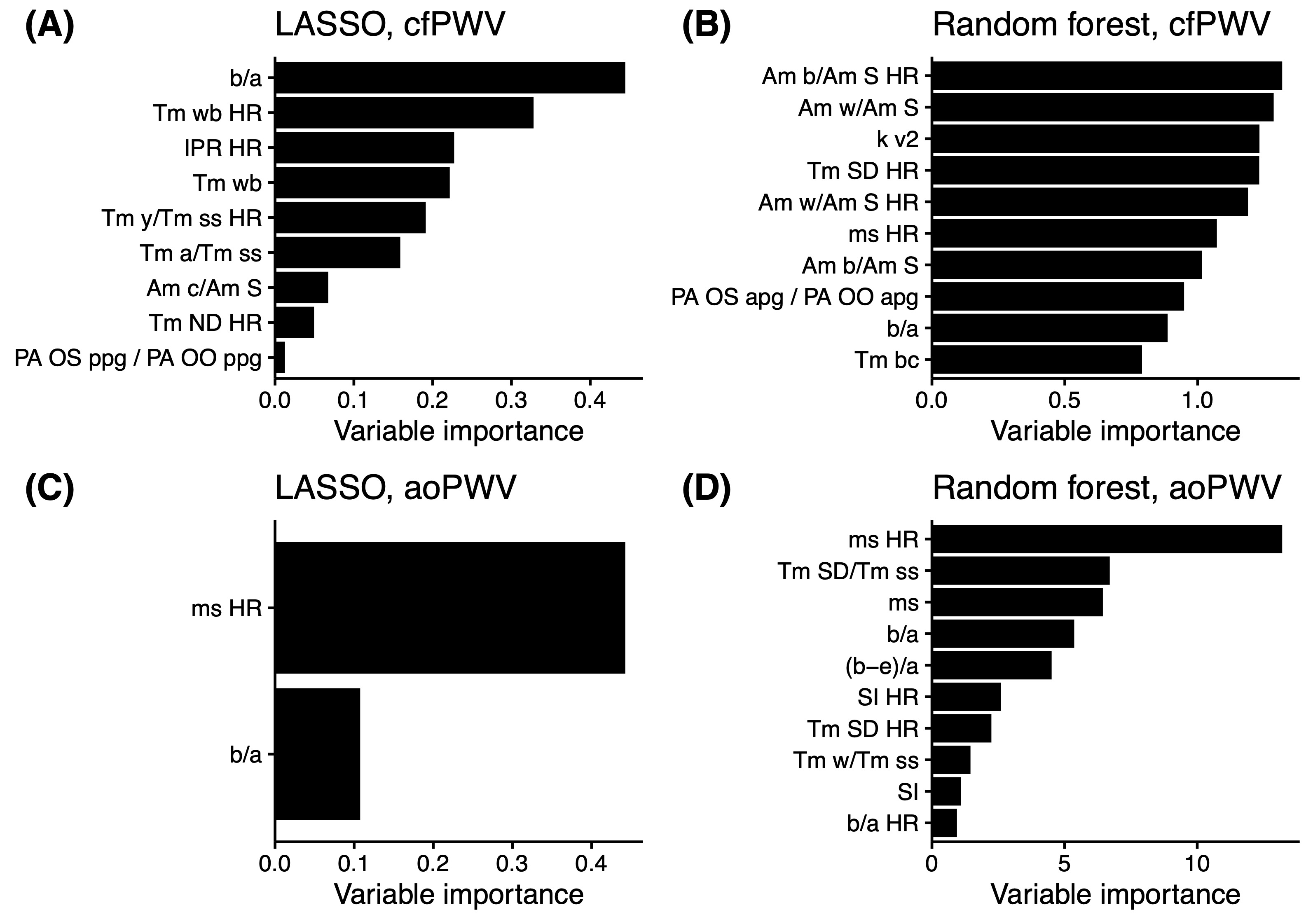
**

**Figure S1.** Variable importance scores from modelling with the baseline feature set. In (A) LASSO and (B) random forest to predict cfPWV, and in (C) LASSO and (D) random forest to predict aoPWV. The scores use relative scales, and up to ten features are shown per model. cfPWV indicates carotid-femoral pulse wave velocity (using SphygmoCor); aoPWV, aortic pulse wave velocity (using Arteriograph); LASSO, least absolute shrinkage and selection operator; HR, adjusted for heart rate. For PPG features, please see Supplemental Table S1 for details.


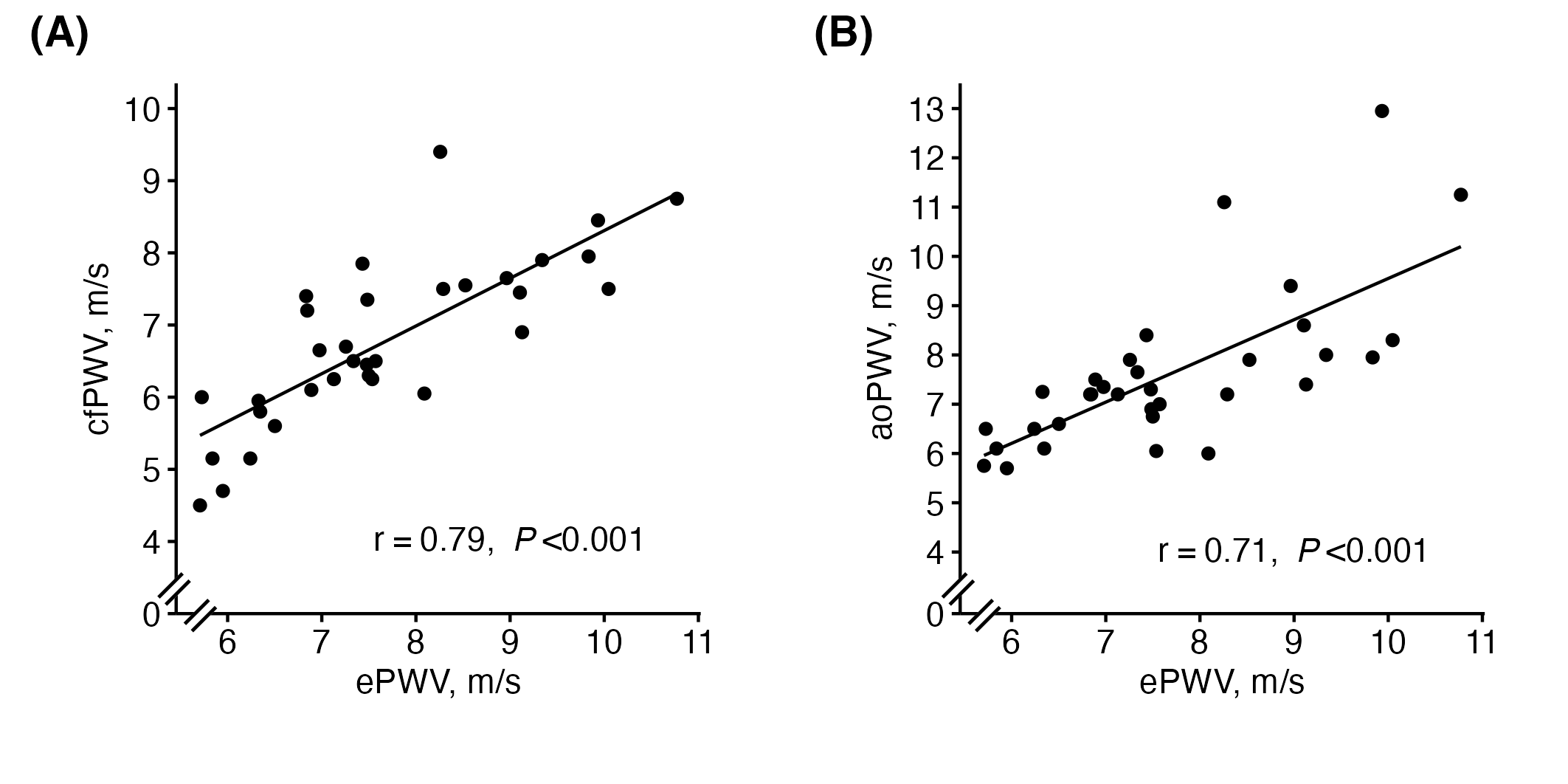


**Figure S2.** Estimated pulse wave velocity in relation to (A) cfPWV and (B) aoPWV. Pearson correlation analysis with r coefficient and *P* value for each scatter plot. The lines represent simple linear regression lines. cfPWV indicates carotid-femoral pulse wave velocity (using SphygmoCor); aoPWV, aortic pulse wave velocity (using Arteriograph); ePWV, estimated pulse wave velocity (based on an equation using age and mean blood pressure).


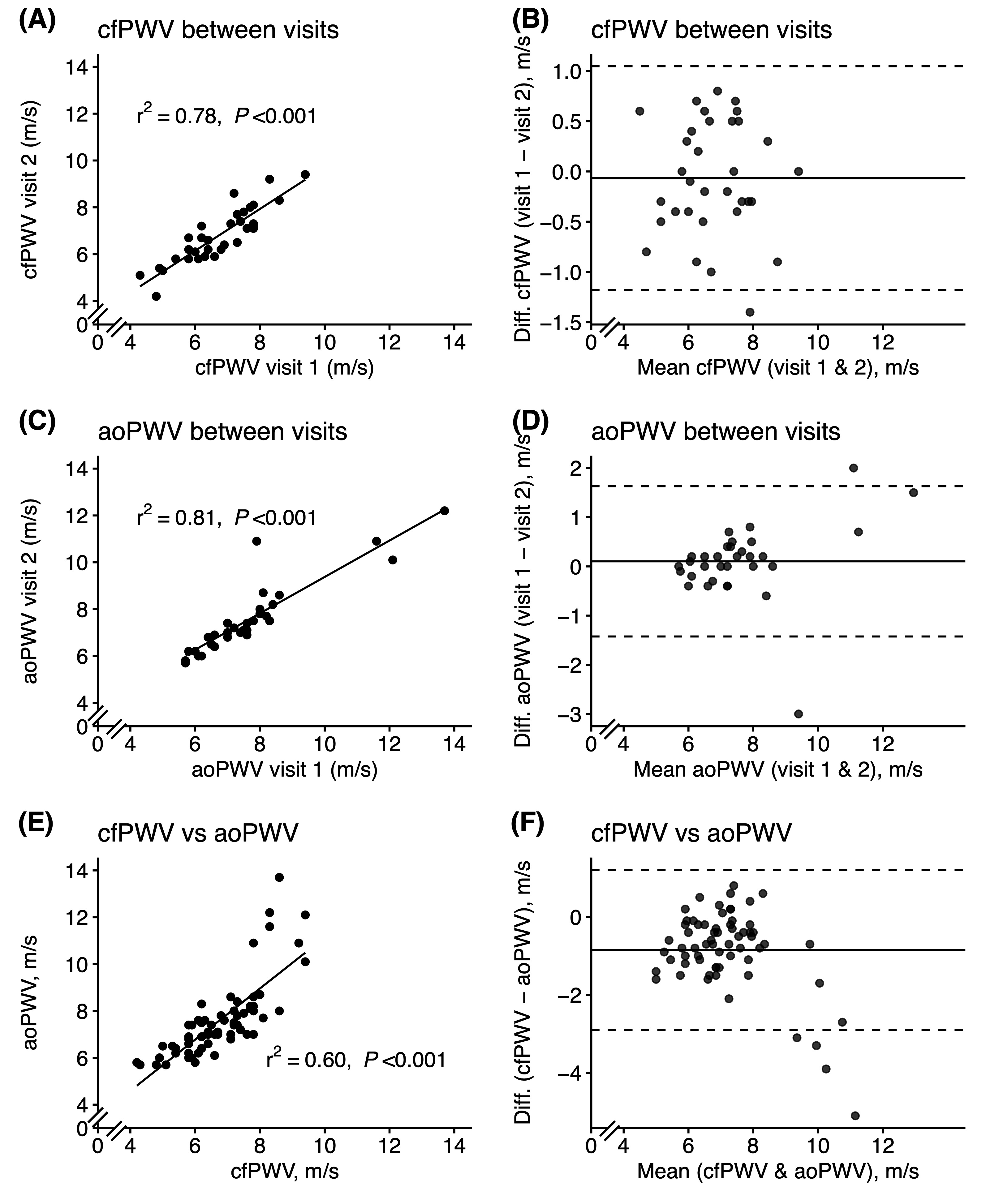

**Figure S3.** Reference equipment repeatability and agreement. (A) shows the correlation for cfPWV between visits with (B) corresponding Bland-Altman plot and (C) the correlation for aoPWV between visits with (D) corresponding Bland-Altman plot. (E) shows the correlation between cfPWV and aoPWV and (F) the corresponding Bland-Altman plot. For scatterplots, the solid line represents a simple linear regression line. For Bland-Altman plots, the solid line represents the bias (mean error) and the dashed lines limits of agreement, which is 1.96 x the standard deviation of the errors. r^2^ indicates the coefficient of determination for the correlation; cfPWV, carotid-femoral pulse wave velocity (using SphygmoCor); aoPWV, aortic pulse wave velocity (using Arteriograph).

**
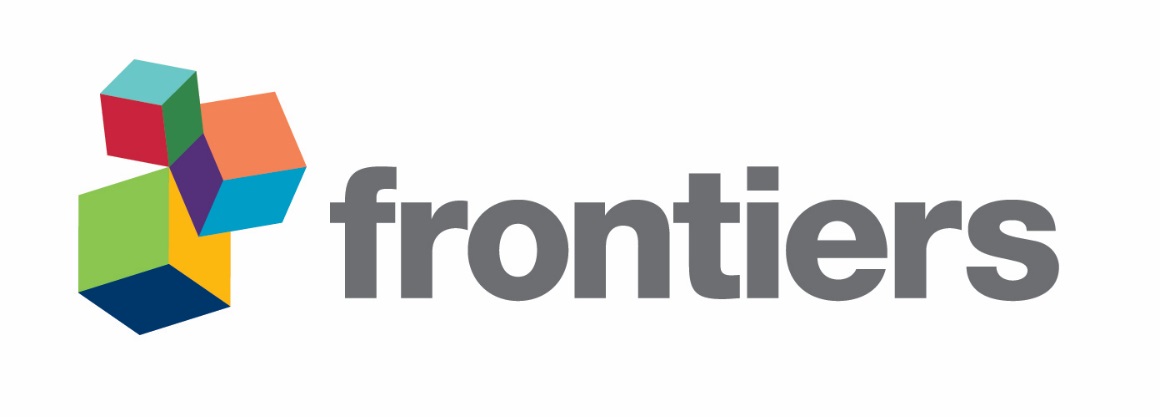
**
